# Limited Practical Utility of Liquid Biopsy in the Treated Patients with Advanced Breast Cancer

**DOI:** 10.3390/diagnostics10080523

**Published:** 2020-07-28

**Authors:** Anna Niwinska, Aneta Bałabas, Maria Kulecka, Anna Kluska, Magdalena Piątkowska, Agnieszka Paziewska, Kazimiera Pyśniak, Wojciech Olszewski, Michał Mikula, Jerzy Ostrowski

**Affiliations:** 1 Department of Breast Cancer and Reconstructive Surgery, Maria Sklodowska-Curie National Research Institute of Oncology, 02-781 Warsaw, Poland; annaniwinska@gmail.com; 2 Department of Genetics, Maria Sklodowska-Curie National Research Institute of Oncology, 02-781 Warsaw, Poland; abalabas@coi.waw.pl (A.B.); mkulecka@cmkp.edu.pl (M.K.); akluska@coi.waw.pl (A.K.); mpiatkowska@coi.waw.pl (M.P.); agapaziewska@poczta.onet.pl (A.P.); pysniak@coi.waw.pl (K.P.); michal.mikula@pib-nio.pl (M.M.); 3 Department of Gastroenterology, Hepatology and Clinical Oncology, Centre of Postgraduate Medical Education, 02-781 Warsaw, Poland; 4 Department of Pathology and Laboratory Medicine, Maria Sklodowska-Curie National Research Institute of Oncology, 02-781 Warsaw, Poland; wojciech.olszewski@pib-nio.pl

**Keywords:** breast cancer, liquid biopsy, cf-DNA, NGS sequencing, Ion Torrent

## Abstract

Recently, liquid biopsy has emerged as a tool to monitor oncologic disease progression and the effects of treatment. In this study we aimed to determine the clinical utility of liquid biopsy relative to conventional oncological post-treatment surveillance. Plasma cell-free (cf) DNA was collected from six healthy women and 37 patients with breast cancer (18 and 19 with stage III and IV tumors, respectively). CfDNA was assessed using the Oncomine Pan-Cancer Cell-Free Assay. In cfDNA samples from patients with BC, 1112 variants were identified, with only a few recurrent or hotspot mutations within specific regions of cancer genes. Of 65 potentially pathogenic variants detected in tumors, only 19 were also discovered in at least one blood sample. The allele frequencies of detected variants (VAFs) were <1% in cfDNA from all controls and patients with stage III BC, and 24/85 (28.2%) variants had VAFs > 1% in only 8 of 25 (32%) patients with stage IV BC. Copy number variations (CNVs) spanning *CDK4*, *MET*, *FGFR1*, *FGFR2*, *ERBB2*, *MYC*, and *CCND3* were found in 1 of 12 (8%) and 8 of 25 (32%) patients with stage III and IV tumors, respectively. In healthy controls and patients without BC progression after treatment, VAFs were <1%, while in patients with metastatic disease and/or more advanced genomic alterations, VAFs > 1% and/or CNV were detected in approximately 30%. Therefore, most patients with stage IV BC could not be distinguished from those with stage III disease following therapy, based on liquid biopsy results.

## 1. Introduction

Circulating cell-free DNA (cfDNA) comprises double-stranded nucleic acid fragments released into body fluids as a result of rupture or apoptosis and necrosis of normal and diseased cells [1,2]. Apoptosis generates cfDNA fragments corresponding to the size of the DNA wrapped around a nucleosome (approximately 145–180 bp, or multiples thereof), whereas necrosis gives rise to irregular and longer cfDNA fragments (up to 21 kb) [3]. Analyses of circulating tumor-derived DNA (ctDNA) in cancer patients are considered an alternative to conventional imaging [4].

Distinguishing between ctDNA and the wild-type cfDNA representing normal cells depends on evaluation of somatic mutations, including single-nucleotide variations (SNVs), small insertions and deletions (indels), gene fusions, and gene amplifications; however, somatic mutations are not only associated with neoplastic cells but are also found in morphologically normal cells; for example, Yizhak et al. recently reported the detection of various somatic mutations in 29 different normal tissue types [5]. While most mutations were found in clones without evidence of cancer pathology, a few were located in genes that could be implicated in cancer. Because normal tissues may have more mutations than previously thought, and the amount of somatic mutations increases with age [5], such alterations of non-cancer origin may significantly influence interpretation of liquid biopsy results. Hence, it is important to distinguish the presence of mutated clones in normal tissues from patient-specific somatic founder mutations shed from cancers. To achieve this, putative founder somatic mutations present in all cancer cells can be identified using multisite exome sequencing; however, obtaining multiple biopsies may be clinically challenging. Furthermore, subclonal mutations uncovered from a single tissue biopsy may be lost during the clinical course of treatment.

The half-life of cfDNA in circulating blood varies from several minutes to several hours and is affected by various pathological conditions, including tissue damage and regeneration [6,7,8,9]; however, the exact mechanisms underlying cfDNA accumulation remain unclear. While cfDNA levels are lower in healthy individuals (range, 0–100 ng/mL of blood) than in patients with cancer (range, 0–1000 ng/mL of blood) [10], ctDNA represents only a small proportion of total cfDNA. A 4-mL plasma aliquot isolated from a 10-mL blood sample of a patient with early-stage cancer typically yields only 20 ng of cfDNA, significantly less than that obtained from patients with metastatic tumors, and after neoadjuvant therapy ctDNA is undetectable in more than 90% of patients [11,12]. Nevertheless, as recently described by McDonald et al. [11], improved analytical sensitivity and quantitative precision of ctDNA analysis allowed detection of ctDNA in 100% of patients with early and locally advanced breast cancer (BC) before treatment, with a median pretreatment ctDNA allele fraction (AF) of 0.11%, and median ctDNA AF levels after neoadjuvant therapy of 0.017% and 0.003% in patients with residual disease and pathological complete response, respectively.

Hence, the spectrum of mutations detected in liquid biopsy samples represents a combination of DNA variants from both normal cell populations and cancer, which can be affected by endogenous and exogenous processes, including radio- and chemotherapy. In this study, using a targeted cancer panel, we compared the post-treatment tumor mutation profiles in patients with stage III and IV BC to evaluate the clinical utility of cfDNA genetic profiling.

## 2. Materials and Methods

### 2.1. Patients

In this prospective cohort study, 37 patients with BC were enrolled from January 2017 to September 2017. In addition, cfDNA from six healthy women was assessed. All subjects provided written informed consent, prior to participation, and the study complied with the Declaration of Helsinki. Patients with BC were divided into two groups according to clinical stage: stage III (locally advanced; *n* = 18) and stage IV (metastatic; *n* = 19).

#### 2.1.1. Patients with Stage III BC

Among 18 patients with stage III (locally advanced) BC, median age was 62 years (range, 35–86 years). Seventeen had non-luminal HER2-positive BC (ER, PR-, HER2+) and one had triple-negative BC (TNBC; ER-, PR-, HER2-). All 18 patients underwent neoadjuvant systemic treatment, surgery, and radiation therapy, while 17 received anti-HER2 targeted therapy, before blood samples were collected. Core biopsies of the primary tumor were performed before treatment in all patients, and tumor specimens collected after mastectomy were also assessed in six patients, to compare changes following neoadjuvant therapy. The median time between collection of tumor tissue sample sent for next-generation sequencing (NGS) (core biopsy before treatment) and collection of plasma sent for ctDNA NGS was 10 months. The demographic and clinical characteristics of cancer, therapy, and recurrence for the 18 patients with stage III disease are summarized in Table 1.

#### 2.1.2. Patients with Stage IV BC

The second group consisted of 19 patients with stage IV disease (distant metastases and/or loco-regional recurrence) at commencement of blood sampling and a median age of 51 years (range, 34–81 years). This group comprised 6 patients with TNBC (ER-, PR-, HER2-) and 13 with non-luminal HER2-positive BC (ER-, PR-, HER2+). The characteristics of these patients, including systemic treatment used during the period of blood sample collection, are presented in Table 2. All patients underwent core biopsy of the primary tumor before treatment and an additional tumor specimen collected after mastectomy was assessed for six patients. The median time between collection of tumor sample sent for tissue NGS (core biopsy before treatment) and collection of plasma sent for ctDNA NGS for these 19 patients with metastatic disease was 14 months.

### 2.2. Blood Sample Collection

Blood samples were taken every 3 months over a period of 9 months. Four blood sample measurements were obtained from 17 (94%) of 18 patients with stage III BC. In the group of 19 patients with stage IV disease, four, three, and two blood samples were collected from 12 (63%), 1 (5%), and 6 (32%) patients, due to patient refusal (1 patient) or death (6 patients). All patients were observed during blood collection (September 2017 to May 2018) and then followed-up to July 2019.

### 2.3. Sequencing of Tumor Tissue DNA

Formalin-fixed paraffin-embedded (FFPE) tissue samples (*n* = 35) obtained from core tumor needle biopsies and resected tumor samples (*n* = 6) were available; macro-dissected samples from these specimens with tumor cell fractions > 70% were subjected to analyses. DNA was extracted using a QIAamp DNA Mini Kit (Qiagen, Hilden, Germany), according to the manufacturer’s instructions. DNA concentration was measured fluorescently using a Qubit instrument (Thermo, Waltham, MA, USA), following the manufacturer’s instructions, and stored at −20 °C. DNA was subjected to Ion AmpliSeq Comprehensive Cancer Panel library preparation, which allows analysis of the coding regions of 409 oncogenes and tumor suppressor genes, and libraries were sequenced using the Ion Proton system (Thermo, Waltham, MA, USA).

### 2.4. Sequencing of Circulating Free Tumor DNA

Blood samples were collected in 10 mL Cell-Free DNA BCT tubes (Streck, La Vista, NE, USA) to ensure high-quality preservation of cfDNA for up to several days, even at room temperature. cfDNA was isolated from plasma using a QIAmp Circulating Nucleic Acid Kit (Qiagen Hilden, Germany) and then prepared for sequencing using an Oncomine Pan-Cancer Cell-Free Assay (Thermo, Waltham, MA, USA). This commercial platform uses cfDNA to identify genomic alterations among 272 amplicons from 52 cancer-related genes (*AKT1, ALK, APC, AR, ARAF, BRAF, CCND1, CCND2, CCND3, CDK4, CDK6, CHEK2, CTNNB1, DDR2, GNA11, EGFR, ERBB2, ERBB3, ERG, ESR1, ETV1, FBXW7, FGFR1, FGFR2, FGFR3, FGFR4, FLT3, GNAQ, GNAS, HRAS, IDH1, IDH2, KIT, KRAS, MAP2K1, MAP2K2, MET, MTOR, MYC, NRAS, NTRK1, NTRK3, PDGFRA, PIK3CA, PTEN, RAF1, RET, ROS1, SF3B1, SMAD4, SMO,* and *TP53*), including copy number variations (CNVs) of 12 genes (*CCND1, CCND2, CCND3, CDK4, CDK6, EGFR, ERBB2, FGFR1, FGFR2, FGFR3, MET,* and *MYC*), and 13 gene fusions involving *ALK, BRAF, ERG, ETV1, FGFR1, FGFR2, FGFR3, MET, NTRK1, NTRK3, RET,* and *ROS1*. This commercial platform for sequencing barcoded libraries enables identification of DNA variants in selected regions of 52 key driver genes at allele frequencies > 0.1%.

### 2.5. Post-Sequencing Data Analyses and Variant Calling

Raw reads were processed using the Torrent Suite analysis pipeline and mapped to human genome assembly hg19 using TMAP. Variant calls were made with Torrent Variant Caller (version 5.0 for FFPE and version 5.8 for cfDNA), using default parameters for somatic variants and cfDNA variants, respectively. Additionally, a custom IonReporter pipeline for paired somatic samples was used to identify variants shared between FFPE biopsy and mastectomy samples. Variants called from FFPE samples were filtered using bcftools (version 1.3; parameters used available in Online Resource: Supplementary Table S1). Annotation of variants and prediction of their consequences for mature proteins were conducted using Variant Effect Predictor (version 95, Ensembl Project, United Kingdom), Cravat version 4.3, and Oncomine version 4.2. In FFPE samples only rare (i.e., with minor allele frequency < 2%) and non-synonymous variants were considered in further analyses. Of these variants, potential driver mutations were chosen based on possessing one of the following criteria:

1. CHASM p-value after FDR correction < 0.1 for either variant or gene.
2. Missense mutation present in an oncogene with a PHRED CADD score > 20.
3. Nonsense or frameshift mutation present in a tumor suppressor gene.
4. Missense or splice site mutation with a PHRED CADD score > 20 in a tumor suppressor gene.
5. Mutation designated as pathogenic using Oncomine.
6. Single nucleotide variant in the following genes relevant to BC: *AKT1*, *FBXW7*, *CCND1*, *FGFR1*, *EGFR, KRAS*, *ERBB2*, *PIK3CA*, *ERBB3*, *SF3B1*, *ESR1*, and *TP53*, with a PHRED CADD score > 20.

cfDNA samples were also analyzed using the IonReporter analysis pipeline (version 5.10), and SNVs, indels, gene fusions, and copy number amplifications within a panel of 52 selected cancer-related genes are reported. Variant allele frequency (VAF) is reported as the percentage of mutated DNA variant divided by total cfDNA molecules at a given genomic position.

## 3. Results

At the time of recruitment, 18 and 19 patients received primary diagnoses of stage III (locally advanced) and stage IV (metastatic) BC. Of these, six patients with stage III disease progressed to stage IV during clinical follow-up. Of the 37 patients, four blood samples were taken every 3 months in the 9-month period from 29 (78%). Fewer blood samples were collected from 8 patients, three from 2 (5%) and two from 6 (16%), due to patient refusal (one case) or death.

### 3.1. Sequence Data from Tumor Tissue DNA Samples

At least one FFPE (Formalin-Fixed Paraffin-Embedded) tumor tissue sample was available from 35 patients. The median mapped read count for the Ion AmpliSeq Comprehensive Cancer Panel data was 17,389,499, with a median coverage of 1094 and median percentage of bases with coverage > 100× of 97.39%. Each read was assigned to its respective amplicon to conduct mutation calling and to estimate mutant allele frequencies.

We identified 14,414 unique variants across 409 genes included in the Comprehensive Cancer Panel, ranging from 812 to 9560 variants per tumor sample (Online resource: Supplementary Table S2). Among these, 4215 were considered driver mutations, according to criteria described in the materials and methods section. Variants likely to be pathogenic were subjected to further analyses. The median numbers of pathogenic variants in patients with stage III and IV disease were 38.5 (range, 23–81) and 35 (range, 21–3322), respectively, which did not represent a significant difference. When analyses were confined to the sequences covered by the Oncomine Pan-Cancer Cell-Free Assay, 133 variants were detected across 34 genes. Of these, 61 were considered pathogenic or likely pathogenic, with a median of one variant per subgroup; ranges, 0–2 in stage III and 0–27 in stage IV samples (no significant difference).

**Figure 1:**
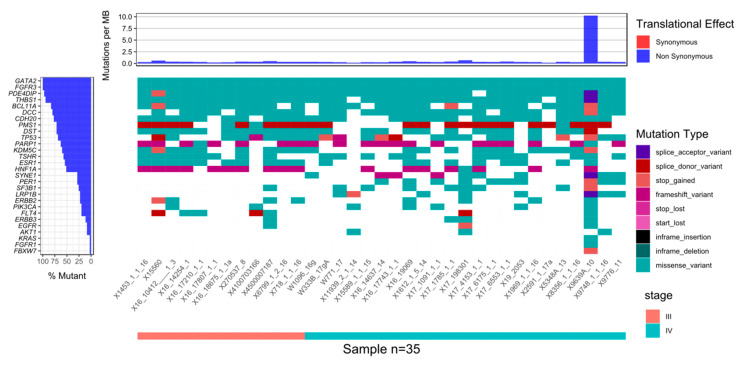
Waterfall plot of variants in most mutated genes as well as genes in which the presence of variants differentiated between stages III and IV samples, and genes relevant to BC (breast cancer). Only variants chosen according to the criteria described in the materials and methods section are shown and only samples from biopsy samples are included.

Genes mutated in at least half of tissue samples from patients in both disease stage subgroups included *GATA2*, *FGFR3*, *THBS1*, *DCC*, *BCL11A*, *CDH20*, *PMS1*, *TP53*, *DST*, *PARP1*, *PDE4DIP*, *KDM5C*, and *TSHR* (Figure 1 and Online resource: Supplementary Table S3).

HNF1A, ESR1, and FLT4 were mutated significantly more frequently in tumor tissue samples obtained from patients with stage III BC, while SYNE1, PER1, and LRP1B were mutated significantly more frequently in stage IV BC (Table 3).

In patients with samples both from the biopsy and mastectomy, the percentages of shared variants ranged from 86% to 88%, with the exception of one sample with a concordance of 44%.

### 3.2. Sequence Data from Plasma DNA Samples

cfDNA was successfully extracted from all 37 enrolled patients and 6 healthy controls, with concentrations ranging from 2.8–349.6 ng/mL of plasma (median, 10.06 ng/mL) and 4.16–6.27 ng/mL of plasma (median, 5.87 ng/mL) in patients and controls, respectively. While cfDNA levels were higher in plasma from patients with BC than in plasma from healthy women (Wilcoxon rank sum test; *p* = 0.0003, cfDNA levels did not differ between patients with stage III (range, 3.28–25.6 ng/mL; median, 8.05 ng/mL) and IV (range, 2.8–349.6 ng/mL; median, 10.88 ng/mL) disease (*p* = 0.09, mixed-effects model).

Using the Oncomine Pan-Cancer Cell-Free Assay (Thermo, Waltham, MA, USA) target enrichment kit, the read counts from all cfDNA samples were >15 million. Median sequencing depth was 19,046,313 mapped reads, with median average coverage 69,777.5, and median molecular coverage 2240.

When raw reads from cfDNA samples were processed using Torrent Variant Caller software, 1120 variants were identified; of these, 154 variants were detected in healthy controls and 1112 were exclusively found in plasma from patients with BC; of the latter, 692 variants were non-synonymous. The median number of alterations observed in each sample for patients with stage III tumors was 39 (range, 26–103), with a corresponding value of 38 (range, 21–205) for patients with stage IV disease; the difference was not statistically significant. Of the 1112 alterations detected, 86% were private variants. Of 61 potentially pathogenic variants present in FFPE samples, 18 (29.5%) were discovered in at least one blood sample and 14 were also detected by IonReporter software (Table 4); only two were discovered in patients with stage III disease.

Annotation of IonReporter results using Oncomine revealed 1–6, 1–11, and 1–4 potentially pathogenic variants in samples from stage III, stage IV, and healthy controls, respectively. Of these, 12.5% (7/56), 24.4% (29/119), and 6% (1/16) had VAFs (variant allele frequencies) of >1% in stage III, stage IV, and healthy control samples, respectively. After removing variants discovered in healthy controls, no variants of the 29 remaining in stage III patient samples had allele frequencies > 1%, while in stage IV patient samples, 28.2% (24/85) had VAF > 1%; the difference between these proportions was statistically significant (*p* = 0.0004 Fisher’s exact test). Differences in VAFs between all detected variants and pathogenic variants did not differ significantly between stage III and IV patient subgroups, nor did differences between stage III and/or IV patient subgroups and healthy controls. In addition, CNVs affecting seven genes (*CDK4*, *MET*, *FGFR1*, *FGFR2*, *ERBB2*, *MYC*, and *CCND3*) were found in cfDNA samples from patients, but not in those from healthy controls. Of these, CNVs that including *CCND3* were detected in 1 of 12 (8%) patients with stage III BC, and those including all genes were present in 8 of 25 patients (32%) with stage IV BC. High-grade *ERBB2* amplification, defined as ≥6 gene copies, was found in five of seven cfDNA samples. The other most commonly unstable regions were *FGFR1* and *FGFR2* (Table 5).

**Figure 2:**
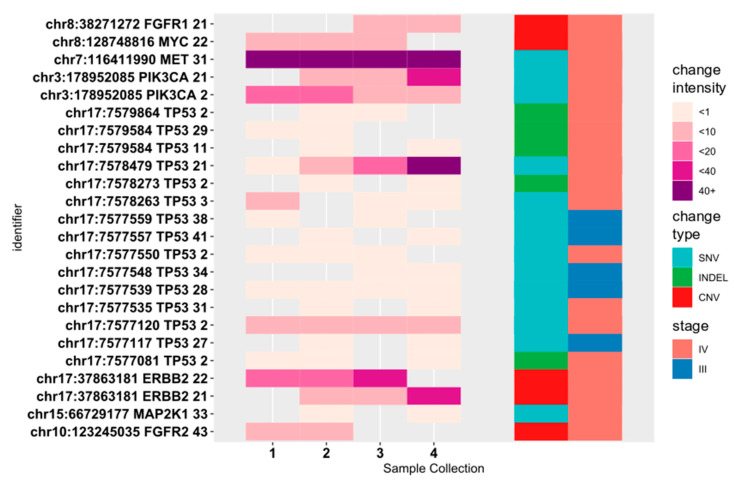
Potentially pathogenic variants found in at least two cfDNA samples from one BC patient. Change intensity is interpreted as allele frequency for SNVs (single nucleotide variant) and indels and as copy number gain for copy number variants.

To check whether the results were consistent across different sample collection times, we also selected mutations observed in at least two samples collected at two different time points from the same patients (Figure 2); variants also present in controls were excluded. Twenty genetic variants and five CNVs were present in more than one sample collected from 15 patients (Online resource: Supplementary Table S4); of these, 14 were in *TP53* while the CNVs were primarily in the *ERBB2* and *FGFR1/2* genes. Five variants were found in stage III patient samples; however, none had VAFs > 1%.

## 4. Discussion

Exome and targeted amplicon parallel sequencing in a patient with metastatic ER-positive and HER2-positive BC, receiving two lines of targeted therapy over three years, identified serial changes in circulating levels of sub-clonal private mutations, which correlated with different treatment responses between metastatic sites; this comparison of biopsy and plasma samples in a single patient showed that ctDNA allows real-time sampling of multifocal clonal evolution [13]. The majority of detected alterations in our study were also private variants, with only a few recurrent or hotspot mutations in cfDNA concentrated in specific regions of cancer-related genes. Of 65 potentially pathogenic variants found in FFPE samples, only 19 (29%) were also discovered in at least one blood sample, likely confirming the influence of post-treatment clonal evolution.

The majority of cfDNA in a patient with cancer is released from normal cells and is mostly apoptotic DNA with a much lower, although markedly varying, proportion of necrotic ctDNA [14,15]. cfDNA concentration can increase due to cancer and cancer-related inflammation themselves; however, it can also result from tissue-damaging therapies, such as chemo- and radiotherapy and surgery [16]. cfDNA levels are low in patients with non-metastatic BC relative to those with metastatic disease [17] and cfDNA levels have been proposed to have diagnostic potential for predicting resistance to therapy and tumor recurrence [18,19]. In this study, we demonstrated that there was a higher cfDNA concentration in plasma samples from patients with BC relative to healthy women; however, cfDNA levels did not differ between patients with stage III (in whom no therapy was conducted during blood drawing) and stage IV (in whom adjuvant treatment was continued) BC.

Although the exact processes involved in the clearance of cfDNA are not fully understood, they lead to a high turnover rate, resulting in rather small amounts of DNA isolated from plasma available for genetic testing [16]. Consequently, the sensitivity and specificity of cfDNA detection depends on the analytical methods used, which can be broadly divided into non-digital PCR-, digital droplet PCR-, and NGS-based platforms [20]. While NGS-based methods can detect low-frequency somatic variants within numerous cancer genes tested simultaneously in highly heterogeneous cfDNA requiring a sensitivity of one in hundreds to thousands, digital PCR can quantitatively detect single-gene variants with a sensitivity > 1 in 10,000. Both methods have some difficulty in selecting true variants from background noise [21,22].

cfDNA sequencing can be performed by targeted and non-targeted techniques. Targeted sequencing is used for detection of known cancer-related loci, while non-targeted whole-genome (WGS) and whole-exome (WES) sequencing are adopted for identification of novel cancer loci with no pre-existing knowledge; however, the higher the number of genes sequenced in a single analysis, the lower the sequencing coverage obtained. Consequently, low depth coverage WGS and WES have limited power to identify alleles with frequencies < 20%, while targeted sequencing can estimate variants with VAFs as low as 1% [16].

Detection of ctDNA in patients with non-metastatic cancer is impeded by limited blood volumes accessible for testing and low levels of cfDNA. In metastatic TNBC, low-coverage genome-wide sequencing determined a tumor fraction among cfDNA in 96.3% of patients and CNVs for 63.9% of patients. A prespecified cfDNA tumor fraction threshold of ≥10% was associated with significantly worse metastatic survival, which remained significant, independent of clinicopathologic factors [23]. Furthermore, digital PCR-based testing identified *TP53* mutations in 75% of patients with nonmetastatic TNBC, and no patient had detectable ctDNA after surgery. While pathological complete response was not correlated with ctDNA detection at any time point, ctDNA positivity after one cycle of neoadjuvant chemotherapy was correlated with shorter disease-free and overall survival [24]. Targeted digital sequencing for multiplex analysis of patient-specific cancer mutations achieved 91% and 53% sensitivity at mutant allele fractions of 3 in 10^4^ and 3 in 10^5^, respectively, analyzing up to 115 mutations per patient in plasma samples from women with stage I–III BC. Thus, this method achieved an up to 100-fold improvement beyond the current limit of ctDNA detection using clinically relevant blood volumes [11]; however, it is unclear if increasing the sensitivity of ctDNA detection will improve understanding of the nature of mutational mosaics detected by liquid biopsy.

In this study, we conducted targeted deep sequencing of cfDNA using an Oncomine assay, testing a panel of cancer-related loci from 52 genes. Molecular barcoding technology included in the Oncomine assay allows identification of variants with increased accuracy and VAF > 0.1%. Finally, we identified hundreds of variants in both patients with BC and healthy controls. In all controls and patients with stage III BC, the allele frequencies of detected variants did not exceed 1%, while in 8 of 25 (32%) patients with stage IV BC, 28.2% (24/85) variants had VAFs > 1%. Thus, this highly sensitive assay allowed the detection of potentially pathogenic variants, even in healthy controls, while shifting the VAF detection threshold above 1% reduces the sensitivity, but increases the specificity, of testing. Notably, however, the identification of variants with VAFs < 1% in the pool of cfDNA approximates a detection level similar to the NGS error rate. Unfortunately, no post-sequencing data analysis allows a clear definition of false positives among selected variants with VAFs < 1%.

Sequencing also facilitates detection of CNVs and chromosomal abnormalities, which provide more unambiguous results that are easier to interpret. In our study, only CNVs including *CDK4*, *MET*, *FGFR1*, *FGFR2*, *ERBB2*, *MYC*, and *CCND3* were found in cfDNA. Of these, the CNV of *CCND3* was found in 1 of 12 (8%) patients with stage III BC, while VAFs in all genes listed above were detected in 8 of 25 patients (32%) with stage IV BC. The most commonly unstable regions were *ERBB2/Her2* and *FGFR1/2*. Approximately 20% of BCs harbor *ERBB2/Her2* gene amplification [25,26], while *FGFR1* and *FGFR2* amplification are reported in around 14% and 4% of patients with BC, respectively [27,28]. Co-amplification of *FGFR1* and *ERBB2/Her2* is associated with less favorable prognosis compared with no amplification or amplification of either gene alone [29]. In our study, four patients harboring CNVs died within nine months of commencement of the investigation and two of them (P21 and P22) had concurrent amplification of the *ERBB2/Her2* and *FGFR1* genes. Notably, CNVs were completely absent from healthy controls and thus may possess higher diagnostic potential that point mutations, particularly since copy number gains in these seven genes were also absent (with the possible exception of *MYC*) from a germline CNV dataset generated by Shaikh et al. based on data from 2026 disease-free individuals [30].

Previous studies suggested that ctDNA testing has potential for real-time monitoring of tumor burden in patients with metastatic disease, with ctDNA mutations associated with targeted therapy response in patients with HER2-positive BC, and endocrine therapy response in patients with ER-positive metastatic BC [4,31,32]. To estimate the clinical utility of liquid biopsy, 69 studies with a total of 5736 BC patients were included in a meta-analysis [33]. Although ctDNA mutations were frequently detected in patients with BC, cfDNA levels and frequencies of ctDNA mutations in *TP53*, *PIK3CA*, and *ESR1* did not differ between patients with early and advanced BC. Furthermore, ctDNA mutations appear to be useful prognostic markers for recurrence and survival and are predictive of auxiliary lymph node metastasis [33]. In patients with resected stage II colon cancer, massive parallel sequencing revealed ctDNA in 8.7% of patients. While there was no clinicopathologic difference between patients with colon cancer with and without detectable ctDNA, postoperative recurrence occurred in 79% of ctDNA-positive patients compared with 10% of ctDNA-negative patients. Moreover, the three-year relapse-free survival rate was 0% for ctDNA-positive patients compared with 90% for ctDNA-negative patients [34]. Our study only partially confirms these earlier observations. Although potentially pathogenic variants with VAFs > 1%, as well as CNV alterations, were mainly detected in patients with stage IV BC, most patients with stage IV disease could not be distinguished from those with stage III, based on our liquid biopsy findings.

ctDNA was first described in 1994 [35,36] and much attention has focused on studies supporting the potential clinical utility of cfDNA evaluation. Indeed, some of these studies supported the hypothesis that an increase in cfDNA level, together with evaluation of tumor mutation profiles and genomic instability using ctDNA, may be useful for diagnosis and quantification of minimal residual disease, as well as for informing therapeutic decisions and monitoring therapy response [2]; however, the clinical utility of liquid biopsy, including its applicability for cancer diagnosis and for monitoring cancer outcomes, has yet to be established [33,37]. ctDNA is primarily distinguished from wild-type cfDNA based on somatic mutations. Nevertheless, somatic mutations are also found in normal cells [38], and most solid tumors do not have a single actionable driver mutation [39], which makes the use of liquid biopsies challenging in clinical practice, especially in the absence of initial information regarding a cancer’s somatic mutations.

## 5. Conclusions

To summarize, the time gap between sampling primary tumor tissues and serial blood draws performed after neoadjuvant therapy, surgery, and adjuvant radio and/or chemotherapy, allowed confirmation of intra- and inter-person genomic heterogeneity, reflecting high levels of post-treatment mutational mosaicism. Serial physical examinations conducted during 1.5 years of post-treatment follow-up did not reveal disease progression in 12 of 18 patients with primary diagnoses of stage III BC. None of them carried plasma pathogenic or likely pathogenic variants with VAFs > 1%. CNVs were detected in 1 of 12 and 8 of 25 patients with stage III and IV BC, respectively. Repeated genomic alterations in samples collected at different times were detected in five patients with stage III disease and 10 patients with stage IV disease. Most pathogenic variants were only found in a single patient (private variants), and CNVs were primarily in the *ERBB2* and *FGFR1/2* genes. In patients without BC progression after treatment, the VAF of detected variants did not exceed 1%, while more advanced genomic alterations (pathogenic mutations with VAF > 1% and/or CNVs) were detected in patients with metastatic disease, particularly those with rapid cancer progression. Nevertheless, the results of liquid biopsy analyses could not distinguish most patients with stage IV BC from those with minimal residual disease at follow-up. Thus, we have found rather limited practical utility of liquid biopsy in the treated patients with advanced breast cancer, and further development of ctDNA detection methods and selection of cancer gene panels, as well uniform plasma collection protocols, are needed before ctDNA-based testing can become standard clinical practice.

## Figures and Tables

**Table 1:** Characteristics of patients with clinical stage III breast cancer (*n* = 18).

Feature	Number of Patients (%)
Initial clinical stage:	
I	−
II	−
III	18 (100)
IV	−
Histological type:	
NST (ductal carcinoma)	17 (94)
Lobular carcinoma	1 (6)
Biological subtype:	
Triple-negative (ER-, PR-, HER2-) *	1 (6)
HER2-positive (ER-, PR-, HER2+)	17 (94)
Preoperative chemotherapy	18 (100)
Type of preoperative chemotherapy **:	
AC × 4 PCL × 12	7 (39)
AC × 4 PCL × 12 + Trastuzumab	2 (11)
TCH	4 (22)
Trastuzumab + PCL × 12	5 (28)
Type of surgery:	
Mastectomy	17 (94)
Breast-conserving treatment	1 (6)
Postoperative radiation therapy	17 (94)
Continuation of trastuzumab to the end of 1 year	17 (94)
Relapse of the disease during blood sampling or further observation ***	6/18 (33)
Location of metastases:	
Lungs	−
Liver	2/6 (33)
Bones	1/6 (17)
Brain	2/6 (33)
Locoregional recurrence	1/6 (17)

* ER, estrogen receptor; PR, progesterone receptor; HER2, receptor for human epidermal growth factor 2; NST, no special type; ** AC, doxorubicin + cyclophosphamide; PCL, paclitaxel; TCH, docetaxel + carboplatin + trastuzumab; *** Of 18 patients initially diagnosed with clinical stage III breast cancer, dissemination of disease was detected in 6 during observation.

**Table 2:** Characteristics of patients with clinical stage IV breast cancer (*n* = 19).

Feature	Number of Patients (%)
Clinical stage at diagnosis:	
I	−
II	6 (32)
III	6 (32)
IV	7 (36)
Histological type:	
NST (ductal carcinoma)	20 (100)
Lobular carcinoma	−
Biological subtype:	
Triple-negative (ER-, PR-, HER2-) *	6 (32)
HER2-positive (ER-, PR-, HER2+)	13 (68)
Location of metastases:	
Lungs	9/19 (47)
Liver	7/19 (37)
Bones	8/19 (42)
Brain	7/19 (37)
Locoregional recurrence	4/19 (21)
Type of treatment of distant metastases during blood sampling:	
Systemic treatment with/without radiation therapy	17/19 (89)
Radiation therapy only	3/19 (11)
Type of systemic treatment during blood sampling:	
Pertuzumab + Trastuzumab + Docetaxel	11/19 (58)
Doxorubicin + Paclitaxel + Trastuzumab	2/19 (11)
Capecytabine	4/19 (21)

* ER, estrogen receptor; PR, progesterone receptor; HER2, receptor for human epidermal growth factor 2; NST, no special type.

**Table 3:** Genes in which the presence of variants differed significantly between stages III and IV patient samples (nominal *p* < 0.05; Fisher’s exact test).

Gene	Stage III	Stage IV	*p*-Value
*SYNE1*	0	10 (43.5%)	0.006942
*PER1*	0	10 (43.5%)	0.006942
*HNF1A*	10 (83.3%)	8 (34.8%)	0.011643
*ESR1*	10 (83.3%)	9 (39.1%)	0.029821
*LRP1B*	0	8 (34.8%)	0.031560
*FLT4*	5 (41.7%)	2 (8.7%)	0.033076

**Table 4:** Allele frequencies of pathogenic variants detected in FFPE also present in cfDNA samples based on Torrent Variant Caller results.

Patient Number	Stage	Gene	Amino Acid/Nucleotide Change	CF				FFPE		IonReporter
11	IV	*PIK3CA*	p.Glu542Lys	0.0	0.3	2.3	0.0	57.5		YES
11	IV	*TP53*	c.652_654dup	0.1	0.5	1.7	0.0	67.1		NO
13	IV	*ERBB2*	p.Ile654Val	40.4	48.6	49.8	43.7	6.3		YES *
13	III	*ROS1*	p.Gly2245Ser	49.4	50.7	49.4	50.0	28.4		YES *
2	IV	*TP53*	p.Arg273His	2.7	5.3	3.4	1.8	0.2		YES
2	IV	*PIK3CA*	p.His1047Arg	11.8	16.2	9.9	4.5	25.7		YES
21	IV	*TP53*	p.Pro151Ala	0.2	1.5	16.1	74.2	60.4		YES
21	IV	*PIK3CA*	p.His1047Arg	0.0	1.4	6.0	22.5	29.5		YES
22	IV	*TP53*	c.559 + 1G > A	21.9	24.2	50.2		13.9		YES *
3	IV	*TP53*	p.Arg196Ter	2.8	0.0	0.1	0.3	34.6		YES
31	IV	*TP53*	p.Cys176Tyr	3.8	1.8	8.7	0.9	52.1		YES
35	IV	*TP53*	p.Arg248Trp	0.0	11.7			35.4	13.1	YES
35	IV	*TP53*	p.Arg181Cys	0.0	18.7			45.8	12.3	NO
35	IV	*PIK3CA*	p.Glu542Lys	0.0	7.6			19.6	5.0	YES
44	IV	*TP53*	p.His178Asp	0.0	0.0	0.0	1.6	3.7		NO
44	IV	*PIK3CA*	p.His1047Arg	0.0	0.0	0.0	2.5	3.6	17.3	YES
6	III	*ROS1*	p.Gly2245Ser	50.2	48.9	51.6	46.1	45.2		YES *
8	IV	*APC*	p.Glu1464Val	50.9	48.9			39.2		NO

CF, molecular allele frequencies in subsequent sample collections; FFPE, allele frequencies in FFPE samples (if samples from mastectomy were available and variant was detected therein, two frequencies are shown); IonReporter, presence and evaluation of variants based on IonReporter results. Variants marked with * were deemed non-pathogenic, according to the Oncomine algorithm.

**Table 5:** Copy number variants detected in cfDNA.

Sample ID	Amplification Rate	Gene	Stage
P27	6p21.1 (41902651–41997949) × 2.34	*CCND3*	III
P1	12q14.1 (58142389–58145535) × 2.31	*CDK4*	IV
P11	7q31.2 (116339104–116435727) × 2.31	*MET*	IV
P11	10q26.13 (123245035–123353779) × 2.32	*FGFR2*	IV
P21 (second sample)	17q12 (37863181–37882971) × 2.76	*ERBB2*	IV
P21 (third sample)	8p11.23 (38271272–38285944) × 2.53	*FGFR1*	IV
P21 (third sample)	17q12 (37863181–37882971) × 8.82	*ERBB2*	IV
P21 (fourth sample)	8p11.23 (38271272–38285944) × 3.73	*FGFR1*	IV
P21 (fourth sample)	17q12 (37863181–37882971) × 31.31	*ERBB2*	IV
P22 (first sample)	8q24.21 (128748816–128753715) × 5.47	*MYC*	IV
P22 (first sample)	17q12 (37863181–37882971) × 15.39	*ERBB2*	IV
P22 (second sample)	8q24.21 (128748816–128753715) × 4.9	*MYC*	IV
P22 (second sample)	17q12 (37863181–37882971) × 15.13	*ERBB2*	IV
P22 (third sample)	7q31.2 (116339104–116435727) × 2.48	*MET*	IV
P22 (third sample)	8p11.23 (38271272–38285944) × 2.66	*FGFR1*	IV
P22 (third sample)	8q24.21 (128748816–128753715) × 9.03	*MYC*	IV
P22 (third sample)	17q12 (37863181–37882971) × 28.96	*ERBB2*	IV
P35	8p11.23 (38271272–38285944) × 2.4	*FGFR1*	IV
P37	6p21.1 (41902651–41997949) × 2.35	*CCND3*	IV
P43 (first sample)	10q26.13 (123245035–123353779) × 3.45	*FGFR2*	IV
P43 (second sample)	10q26.13 (123245035–123353779) × 3.29	*FGFR2*	IV
P44	17q12 (37863181–37882971) × 2.82	*ERBB2*	IV

## Supplementary Materials

Supplementary Materials can be found at https://www.mdpi.com/2075-4418/10/8/523/s1. Supplementary Table S1 Parameters used for filtering of FFPE results, Supplementary Table S2. All changes discovered in FFPE samples after filtering, Supplementary Table S3. Fisher’s exact test results for presence of deleterious variants in genes, Supplementary Table S4. Genetic alterations and CNVs with repeated measurements during sample collection time.

## Abbreviations

BC	Breast cancer
cfDNA	plasma cell-free (cf) DNA
ctDNA	circulating tumor-derived DNA
